# Temporal association between leg movements and respiratory events in patients with obstructive sleep apnea: description and differences between the AASM and WASM scoring criteria

**DOI:** 10.1007/s11325-023-02844-x

**Published:** 2023-05-06

**Authors:** Zhengfei Huang, Frank Lobbezoo, Nico de Vries, Ghizlane Aarab, Antonius A. J. Hilgevoord

**Affiliations:** 1 Department of Orofacial Pain and Dysfunction, Academic Center for Dentistry Amsterdam (ACTA), University of Amsterdam and Vrije Universiteit Amsterdam, Gustav Mahlerlaan 3004, 1081 LA Amsterdam, the Netherlands; 2 Department of Clinical Neurophysiology, OLVG, Amsterdam, the Netherlands; 3 Department of Otorhinolaryngology - Head and Neck Surgery, OLVG, Amsterdam, the Netherlands; 4 Department of Otorhinolaryngology - Head and Neck Surgery, Antwerp University Hospital (UZA), Antwerp, Belgium

**Keywords:** Obstructive sleep apnea, Respiratory event, Leg movement, Temporal association, Scoring criteria

## Abstract

**Purpose:**

To describe the temporal association between leg movements (LMs) and respiratory events in patients with obstructive sleep apnea (OSA), and to quantify the difference in scoring respiratory-related leg movement (RRLM) between the American Academy of Sleep Medicine (AASM) criterion and the criterion recommended by the World Association of Sleep Medicine (WASM).

**Methods:**

Patients with OSA who presented with > 10 LMs of any type per hour of sleep were included in this study. For each participant, RRLMs were scored using both the AASM criterion and the recommended WASM criterion. The occurrence of LMs in relation to respiratory events and the difference in scoring RRLM between the AASM criterion and the criterion recommended by the WASM were quantified.

**Results:**

In 32 patients enrolled, mean age was 48.1 ± 11.0 years and 78% were men. LMs were significantly more frequent after respiratory events, followed by before respiratory events, and were rare during respiratory events (*P* < 0.01). Compared with the AASM criterion, more LMs were classified as RRLMs based on the recommended WASM criterion (*P* = 0.01).

**Conclusion:**

LMs are more frequent after respiratory events than before and during respiratory events, and more LMs are scored as RRLMs based on the recommended WASM criterion than based on the AASM criterion.

## Introduction

Several types of involuntary limb movements may occur during sleep. These movements may be isolated in time and occur in response to external triggers (e.g., noise and light) or seemingly spontaneously. Other limb movements may occur repetitively in series. Periodic limb movement during sleep (PLMS) is characterized by repetitive involuntary flexion/extension of the big toe and ankle, and occasional flexion of the knee and hip [1]. The American Academy of Sleep Medicine (AASM) defines a leg movement (LM) as an increase of ≥ 8 μV in the electromyogram (EMG) voltage of the anterior tibialis above its resting voltage with a duration of 0.5 – 10 s [1]. According to the AASM and the World Association of Sleep Medicine (WASM), to be qualified as a PLMS event, a single limb movement has to occur in a series of at least 4 events with regular intervals of 5 – 90 s [1, 2]. The etiology of PLMS is not fully understood, but available evidence suggests that the occurrence of PLMS is controlled by central nervous system (CNS) pacemakers, which govern rhythmic activities like locomotion and respiration. Dopaminergic dysfunctions may play a role in the occurrence of PLMS [3, 4]. The occurrence of PLMS is often associated with repeated arousals during sleep, which may lead to impaired sleep quality and daytime sleepiness [5, 6]. PLMS may be a side effect of using medications like antidepressants [7]. In addition, PLMS is frequently observed in patients with neurological disorders (e.g., Parkinson’s disease [8]) or other sleep-related disorders (e.g., restless legs syndrome and obstructive sleep apnea [OSA]) [2, 9].

**Fig. 1:**
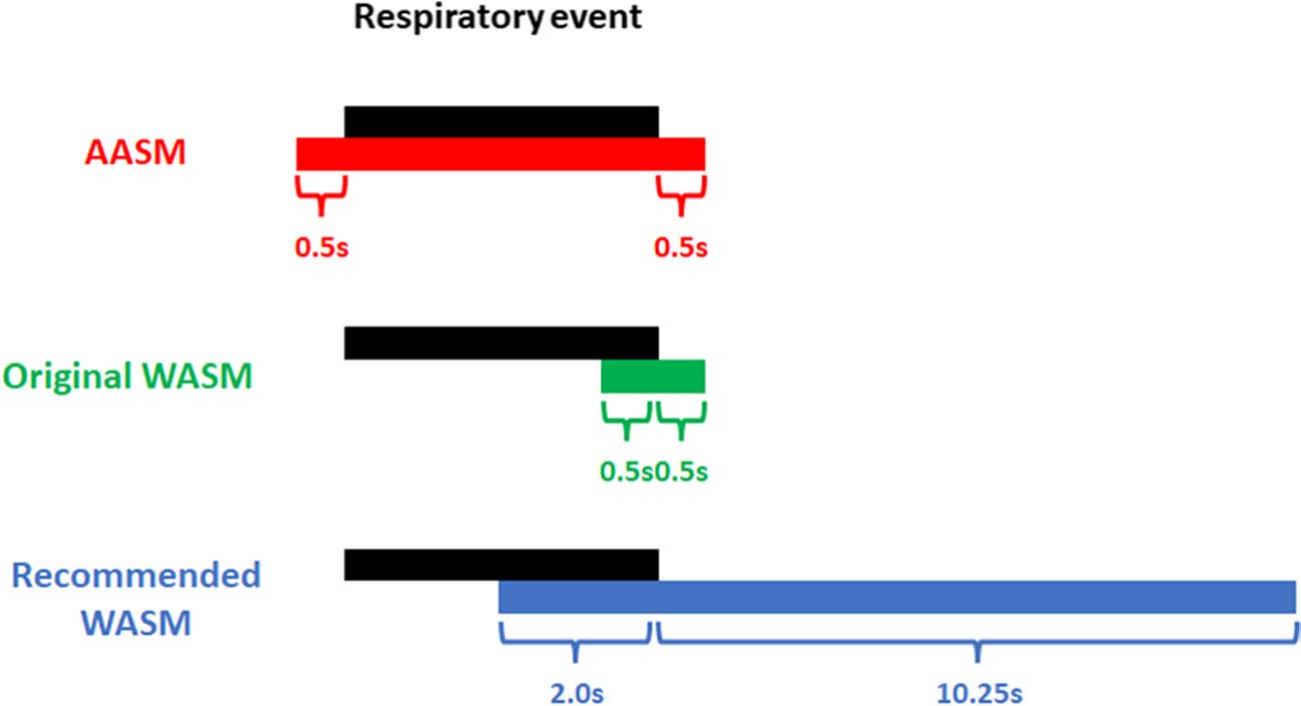
Scoring criteria for RRLM. The black block is a respiratory event, and the red, green, and blue blocks are the time windows to score RRLMs based on the AASM, original WASM, and recommended WASM criteria, respectively. AASM = American Academy of Sleep Medicine; s = seconds; WASM = World Association of Sleep Medicine

OSA is a common sleep-related breathing disorder, which is characterized by recurrent episodes of apnea and/or hypopnea, resulting from complete or partial obstruction of the upper airway during sleep [10]. Repetitive limb movements in OSA may cause a diagnostic problem as they may not only be PLMS but could also be the consequence of respiratory events (viz., apnea, hypopnea, and respiratory effort-related arousal [RERA]). Therefore, in 1993, the American Sleep Disorders Association (ASDA) excluded LMs at the end of respiratory events from being considered as PLMS [11]. In 2006, the WASM first defined LMs that occurred from − 0.5 s to + 0.5 s relative to the end of respiratory events as respiratory-related leg movements (RRLMs) [12]. On the other hand, the AASM defined a time interval from 0.5 s preceding respiratory events to 0.5 s following the respiratory events for RRLMs [1]. However, both RRLM criteria were based on experts’ opinions. In a previous clinical study from Manconi et al., the probability of LM was found to increase during an interval of − 2.0 s to + 10.25 s around the end of respiratory events, rather than at the beginning or middle of respiratory events [13]. Based on this finding, the latest (2016) recommended WASM criterion is to score LMs that occur during a period between 2.0 s before the end and 10.25 s after the end of respiratory events as RRLMs [4]. The AASM still uses the original scoring criterion for RRLM [14] (Fig. 1).

Application of either the WASM or AASM criteria may directly affect LM-related diagnosis and treatment outcomes. The possibility has been suggested that, by using the original WASM and AASM scoring criteria for RRLM, the number of periodic leg movements (PLMs) in patients with OSA is considerably overestimated, while the number of RRLMs is considerably underestimated [13]. If a large amount of RRLMs is misrecognized as PLMs, a patient might be misdiagnosed with periodic leg movement disorder (PLMD). However, recommended treatments for PLMD (e.g., lifestyle changes [15] and medication [16, 17]) will not be effective in reducing RRLMs. On the other hand, the scoring of RRLMs may also overrule potential LM-related diagnoses, such as restless leg syndrome and PLMD, especially in patients with severe OSA. This is because that such patients present with many respiratory events during sleep and many adjacent LMs are consequently scored as RRLMs, even though in nature they may not be driven by respiratory events and just happen to co-occur with respiratory events [18]. To optimally distinguish RRLMs from other LMs, firstly, the temporal association between LMs and respiratory events needs to be further quantified, extending previously reported that LMs are more frequent at the end of respiratory events than at the beginning or middle of respiratory events [13, 19]. In addition, the recommended WASM criterion mainly focuses on LMs at the end of respiratory events, while the AASM criterion scores LMs during respiratory events as RRLMs. The quantitative difference in scoring RRLM between the two criteria is unclear. Therefore, the first aim of the present study was to describe the temporal association between LMs and respiratory events in OSA. We hypothesized that, in addition to the end of respiratory events, LMs can also be commonly observed at the beginning and during respiratory events. The second aim was to quantify the difference in scoring RRLM between the AASM criterion and the recommended WASM criterion. We hypothesized that, compared with the AASM criterion, more LMs will be scored as RRLMs based on the recommended WASM criterion.

## Methods

### Participants

Participants in this retrospective, observational study were selected from among adult patients (> 18 yrs) who visited the Department of Clinical Neurophysiology of the OLVG (Amsterdam, The Netherlands) between July 2020 and December 2021 for snoring and potential OSA. Included patients were those diagnosed with OSA by polysomnography (PSG; apnea-hypopnea index [AHI] > 5 events/hour), presented with > 10 LMs of any type per hour of sleep. All participants provided informed consent for using their data for scientific purposes. This study was approved by the local ethical committee (study number: WO 19.079).

### Polysomnography

Ambulatory PSG recordings (SOMNOscreen Plus, Randersacker, Germany) were performed in accordance with the standard protocol of the hospital. All PSG recordings were manually scored according to the standard criteria (AASM, version 2.6, 2020) [14] by an experienced sleep technician to determine, amongst others, apnea, hypopnea, RERA, and LM. Standard scoring of RRLMs was based on the AASM criteria. LMs that occurred during a period from 0.5 s preceding respiratory events (apnea, hypopnea, or RERA) to 0.5 s following respiratory events were scored as RRLMs. For this study, we also scored RRLMs according to the recommended WASM criterion. To that end, data were exported and reanalyzed using R (version 4.0.5, Vienna University of Economics and Business, Vienna, Austria). A LM was scored as an RRLM if any part of the LM lies within the interval of − 2.0 s to + 10.25 s around the end of a respiratory event [13].

### Visualization

Based on the exported PSG data, hypnograms were constructed using the R package ‘plotly’ (version 4.10.0) [20]. Hypnograms included sleep stage (viz., wake, non-rapid eye movement sleep stage 1–3 [N1-3], and rapid eye movement sleep [REM]), LM (viz., isolated leg movement [ILM; not body position-related], PLM, and RRLM), respiratory event (viz., apnea, hypopnea, and RERA), arousal, and sleep position (viz., upright, supine, left lateral decubitus position, right lateral decubitus position, and prone).

### Quantification

For each respiratory event, the occurrence of LMs was quantified over three time windows relative to the respiratory event (viz., from 20 s [13] before the onset of respiratory event to the onset of respiratory event, from the onset to the end of respiratory event, and from the end of respiratory event to 20 s after the end of respiratory event). Hence, it was possible that a LM is counted both as a LM at the end of the preceding respiratory event and as a LM at the beginning of the following respiratory event. Notably, because a respiratory event can be either longer or shorter than 20 s, to ensure the comparability between the data from these three time windows, the duration of respiratory events and the number of LMs during respiratory events were normalized to 20 s. For example, for a 10 s respiratory event during which three LMs occurred, the duration of the respiratory event and the number of LMs during this respiratory event would be normalized to 20 s by multiplying by two. After normalization, the number of LMs during this respiratory event was six. Both the original data and the normalized data would be used for further statistical analyses. These procedures were repeated for each participant. In addition, for each participant, the number of RRLMs that were identified based on the AASM criterion and the recommended WASM criterion was calculated. All the above-mentioned procedures were performed using R.

### Statistics

In descriptive statistics, nominal and ordinal data were presented as percentages. For continuous data, Kolmogorov–Smirnov test was used to check if the data was normally distributed. Normally distributed data were presented as mean ± standard deviation (SD). To better present the characteristics of the data in this study, non-normally distributed data were presented both as mean ± SD and as median (interquartile range; IQR). In the present study, as multiple respiratory events can be extracted from the recording of a single participant, a multilevel dataset was obtained, i.e., respiratory events (level 1) were nested within individuals (level 2). To adjust for the fact that multiple respiratory events from one participant were not independent of each other, generalized linear mixed model (GLMM) was used to investigate the difference between the number of LMs that occurred before, during, during (normalized), and after respiratory events. To quantify the difference in scoring RRLM between the AASM criterion and the recommended WASM criterion, paired t-test was used for normally distributed data, otherwise Wilcoxon test was used. Significance level was set as 0.05. Analyses were conducted using the IBM SPSS Statistics 27 software package (IBM Corp, Chicago, USA).

## Results

A total of 88 eligible patients visited the Department of Clinical Neurophysiology of the OLVG between July 2020 and December 2021 for snoring and potential OSA. Of these 88 patients, 34 were excluded due to an AHI of less than 5, 19 were excluded due to less than 10 LMs/h, and 3 were excluded due to technically failed PSG. After exclusions, 32 participants were included in the present study. The characteristics of participants are shown in Table 1.

**Table 1:** The characteristics of participants

Characteristics (*n* = 32)		
	Percentage	
Male gender	78 (25/32)	
	Mean ± SD	Median (IQR)
Age (year)	48.1 ± 11.0	48.0 (39.0 – 55.8)
BMI (kg/m2)	29.3 ± 5.5	27.0 (25.3 – 32.0)
AHI (events/hour)	28.3 ± 26.5	20.5 (8.3 – 39.5)
Total leg movement (events/hour)	27.4 ± 14.7	21.5 (15.0 – 39.7)
ILM (events/hour)	12.4 ± 4.6	11.9 (10.0 – 14.6)
PLMI (events/hour)	15.0 ± 13.1	9.9 (5.6 – 23.0)
RRLM (events/hour)	6.3 ± 9.0	2.2 (0.9 – 7.6)

BMI = body mass index; AHI = apnea–hypopnea index; ILM = isolated leg movement; IQR = interquartile range; PLMI = periodic leg movement index; RRLM = respiratory-related leg movement

### Temporal association between LMs and respiratory events

**Fig. 2:**
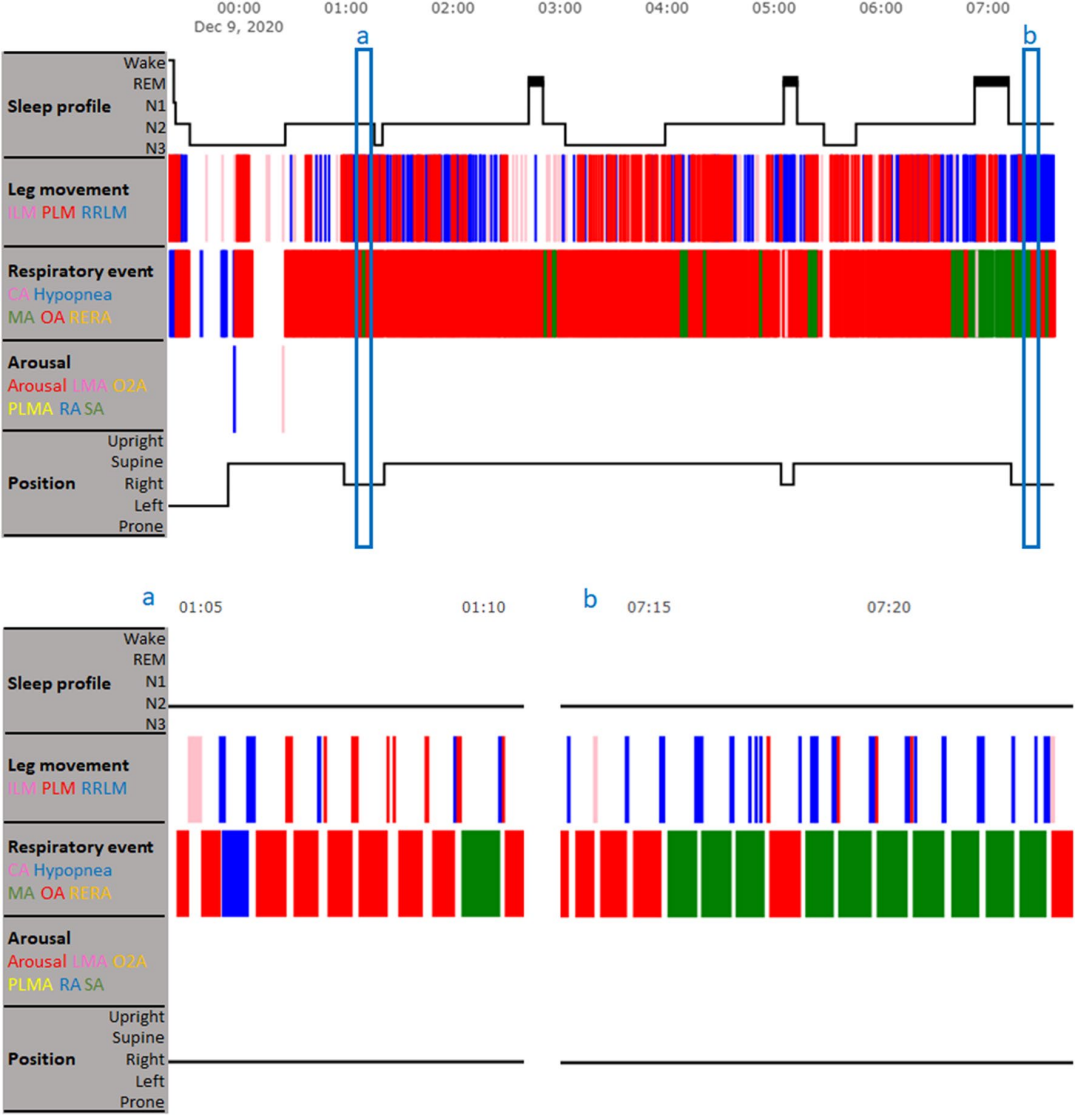
The occurrence of leg movements between two consecutive respiratory events. The upper panel shows a patient’s hypnogram. The two blue boxes a and b are two example time windows, during which leg movements are observed at the end of consecutive respiratory events. The example time windows are enlarged in the lower panel. CA = central apnea; ILM = isolated leg movement; LMA = leg movement arousal; MA = mixed apnea; N1-3 = non-rapid eye movement sleep stage 1–3; O2A = SpO2 arousal; OA = obstructive apnea; PLM = periodic leg movement; PLMA = periodic leg movement arousal; RA = respiratory arousal; REM = rapid eye movement sleep; RERA = respiratory effort-related arousal; RRLM = respiratory-related leg movement; SA = snoring arousal

From the 32 participants, based on the AASM criteria, a total of 7,177 respiratory events were identified and there were 8,205 LMs that were time linked to these respiratory events. Of the 8,205 LMs, 3346 (41%) occurred before respiratory events, 1323 (16%) occurred during respiratory events, and 3536 (43%) occurred after respiratory events. In statistical analyses, it was found that LMs were significantly more frequent after respiratory events, followed by before respiratory events, and were rare during (both with and without normalization) respiratory events (*P* < 0.01; Table 2). It should be noted that the mean and median of LMs in Table 2 refer to each individual respiratory event. Although the difference in the number of LMs before and after each respiratory event seems minor (0.46 and 0.49, respectively), the difference in the total number of LMs before and after respiratory events can be major and significant when patients present with hundreds of respiratory events during sleep, and especially when also considering the high SDs, which are 0.67 and 0.69, respectively. In hypnograms, it can be observed that respiratory events often occurred in a series. As a consequence, a majority of LMs were between two consecutive respiratory events (Fig. 2).

**Table 2:** The difference between the number of leg movements before, during, and after a respiratory event

Leg movements in relation to respiratory events	Number of leg movements		*P* value	
	Mean ± SD	Median (IQR)		
- Before	0.46 ± 0.67	0 (0—1)	< 0.01	*#^
- During	0.19 ± 0.53	0 (0—0)		#^
- During (normalized)	0.15 ± 0.45	0 (0—0)		^
- After	0.49 ± 0.69	0 (0—1)		

* indicates significant difference versus the group “During”; # indicates significant difference versus the group “During (normalized)”; ^ indicates significant difference versus the group “After”. IQR = interquartile range

### Difference in scoring RRLM between the AASM criterion and the recommended WASM criterion

**Fig. 3:**
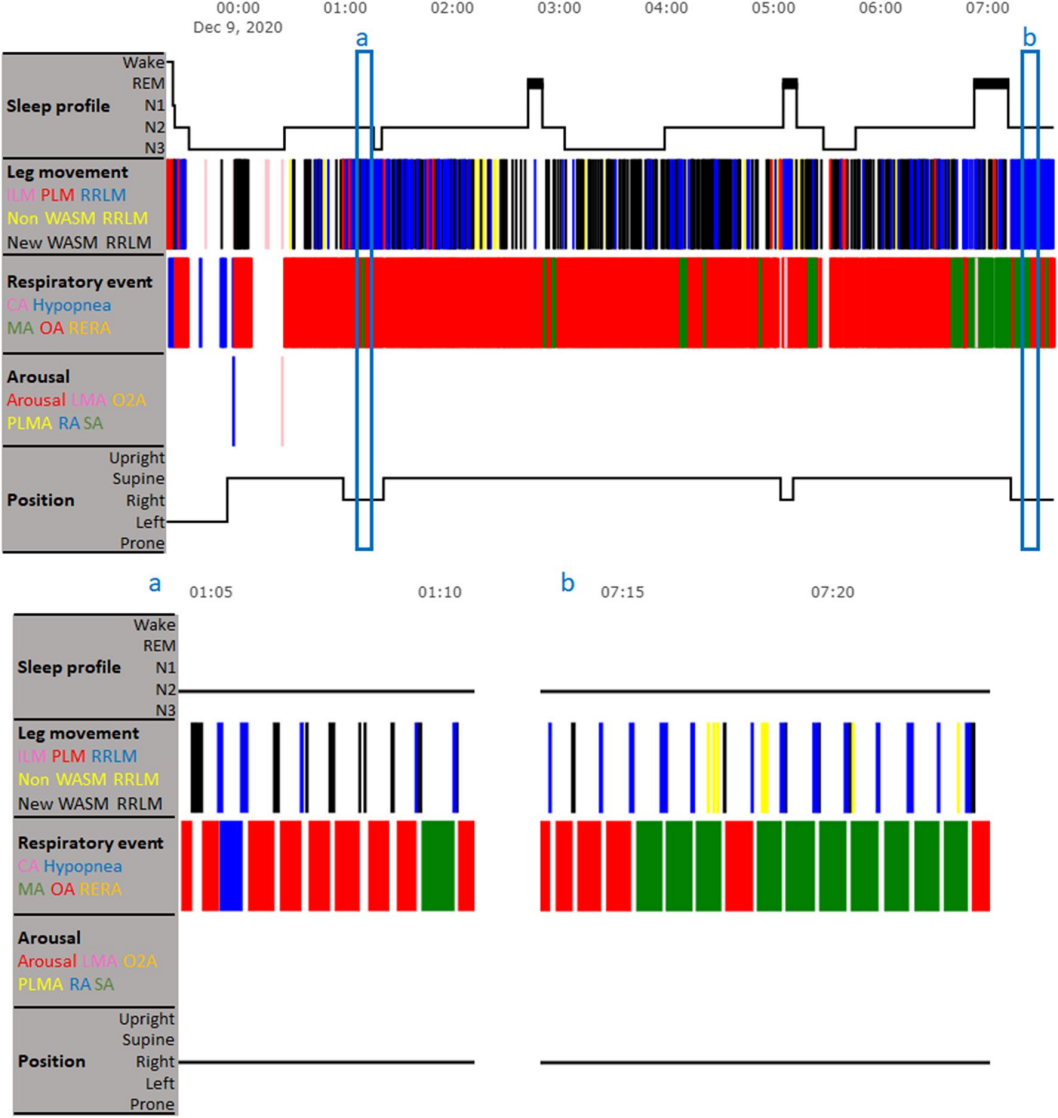
The difference in scoring RRLM between the AASM criterion and the recommended WASM criterion in cases where leg movements mainly occur between consecutive respiratory events. The upper panel shows a patient’s hypnogram. The two blue boxes a and b are two example time windows, during which many AASM criterion-identified PLMs and ILMs between two consecutive respiratory events are scored as RRLMs based on the recommended WASM criterion. The example time windows are enlarged in the lower panel. CA = central apnea; ILM = isolated leg movement; LMA = leg movement arousal; MA = mixed apnea; N1-3 = non-rapid eye movement sleep stage 1–3; Non WASM RRLM = leg movement that is scored as RRLM based on the AASM criterion but is excluded from RRLM based on the recommended WASM criterion; New WASM RRLM = leg movement that is not scored as RRLM based on the AASM criterion but is identified as RRLM based on the recommended WASM criterion; O2A = SpO2 arousal; OA = obstructive apnea; PLM = periodic leg movement; PLMA = periodic leg movement arousal; RA = respiratory arousal; REM = rapid eye movement sleep; RERA = respiratory effort-related arousal; RRLM = respiratory-related leg movement; SA = snoring arousal

**Fig. 4:**
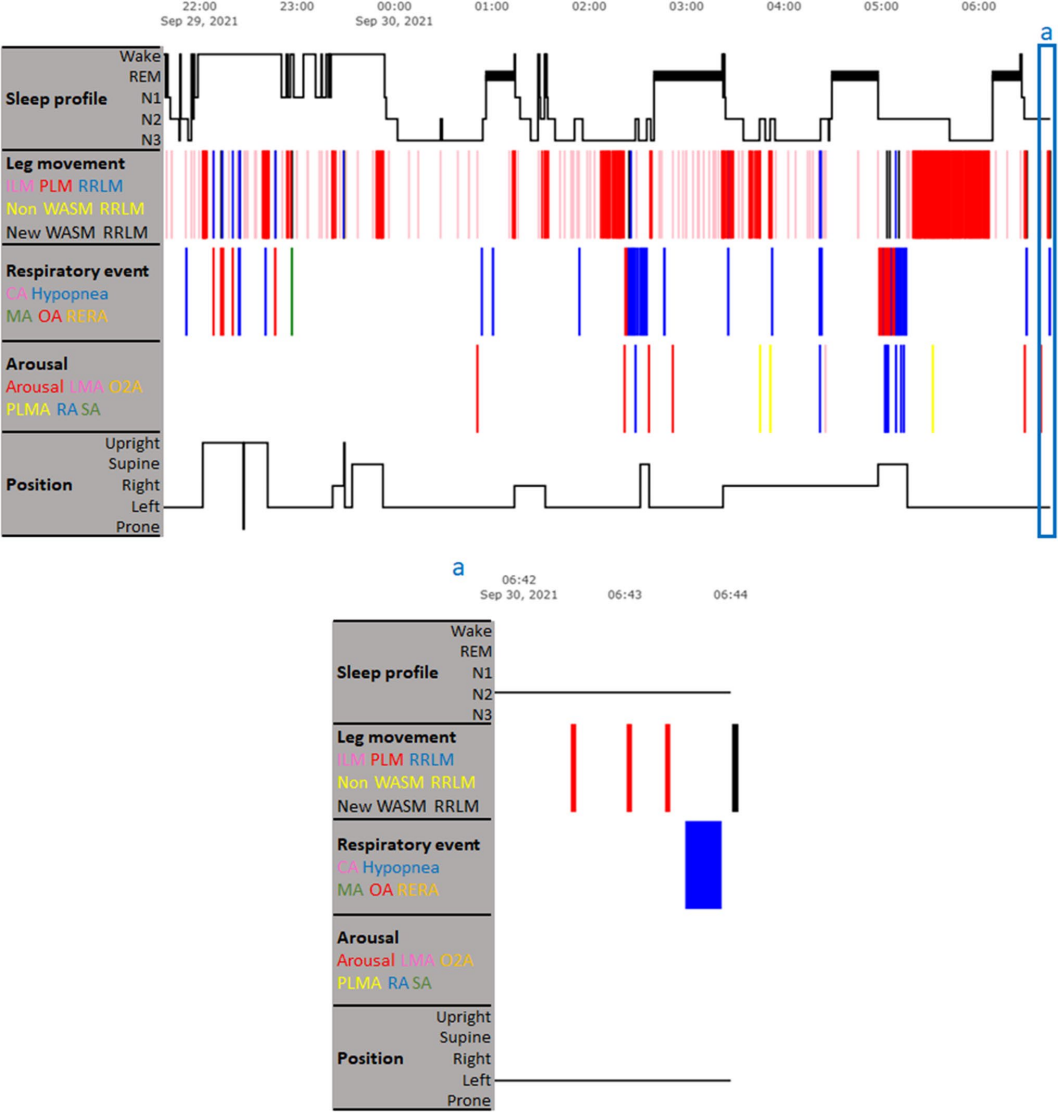
Adopting the recommended WASM criterion disrupts PLMS. The upper panel shows a patient’s hypnogram. The blue box a is an example time window, during which an AASM criterion-identified PLM is scored as a RRLM based on the recommended WASM criterion. As a consequence, the three adjacent AASM criterion-identified PLMs will be scored as three ILMs, because they do not fulfill the criterion to be scored as PLMs anymore. This example time window is enlarged in the lower panel. CA = central apnea; ILM = isolated leg movement; LMA = leg movement arousal; MA = mixed apnea; N1-3 = non-rapid eye movement sleep stage 1–3; Non WASM RRLM = leg movement that is scored as RRLM based on the AASM criterion but is excluded from RRLM based on the recommended WASM criterion; New WASM RRLM = leg movement that is not scored as RRLM based on the AASM criterion but is identified as RRLM based on the recommended WASM criterion; O2A = SpO2 arousal; OA = obstructive apnea; PLM = periodic leg movement; PLMA = periodic leg movement arousal; RA = respiratory arousal; REM = rapid eye movement sleep; RERA = respiratory effort-related arousal; RRLM = respiratory-related leg movement; SA = snoring arousal

Compared with the AASM criterion, more LMs were scored as RRLMs based on the recommended WASM criterion (“New WASM RRLM” in Fig. 3). Most of the LMs that were newly identified as RRLMs based on the recommended WASM criterion were those between two consecutive respiratory events, which were scored as PLMs or ILMs based on the AASM criterion. On the other hand, a small number of LMs that occurred during respiratory events and were scored as RRLMs based on the AASM criterion were excluded from RRLMs based on the recommended WASM criterion (“Non WASM RRLM” in Fig. 3). Statistical analysis confirmed that, in general, compared with the AASM criterion, more LMs were scored as RRLMs based on the recommended WASM criterion (*P* = 0.01; Table 3). A point to note is that some respiratory events did not occur in a series and seemed isolated. In that case, adopting the recommended WASM criterion may hamper PLM quantification. Specifically, if one in a series of four repetitive limb movements is labeled as RRLM, the other three do not validate as PLMs anymore. An example of such case is shown in Fig. 4, where a PLM following an isolated respiratory event was identified as a RRLM based on the recommended WASM criterion. As a consequence, the three LMs adjacent to the new RRLM did not meet the criteria for PLMS and would be scored as three ILMs.

**Table 3:** The difference between the number of RRLMs identified based on the AASM and the recommended WASM criteria

Criteria	Number of RRLMs		*P* value
	Mean ± SD	Median (IQR)	
- The AASM criterion	50.5 ± 70.2	19.5 (8.3 – 56.3)	0.01
- The recommended WASM criterion	90.7 ± 112.1	42.0 (20.3 – 123.0)	

AASM = American Academy of Sleep Medicine; IQR = interquartile range; RRLM = respiratory-related leg movement; WASM = World Association of Sleep Medicine

## Discussion

This study aimed to: i) describe the temporal association between LMs and respiratory events in patients with OSA; and ii) quantify the difference in scoring RRLM between the AASM criterion and the recommended WASM criterion. It was found that LMs were significantly more frequent after respiratory events, followed by before respiratory events, and were rare during respiratory events. In addition, compared with the AASM criterion, more RRLMs were identified based on the recommended WASM criterion.

The first finding of this study is in line with previous studies [13, 19] and is partially confirmed by the second finding. Specifically, compared with the AASM criterion, the recommended WASM criterion shortens the time window to score LMs before and during respiratory events as RRLMs, while it extends the time window to score those at the end of respiratory events as RRLMs. It turned out that, compared with the AASM criterion, significantly more RRLMs were scored based on the recommended WASM criterion, suggesting that LMs are more frequent at the end of respiratory events than before and during respiratory events, and that there is an association between respiratory events and LMs.

Given that respiratory events often occurred in a series, LMs were frequent between two consecutive respiratory events. It can be observed in the present study that, by using the recommended WASM criterion, most of LMs between consecutive respiratory events were scored as RRLMs. This is consistent with the authors’ opinion that when scoring RRLMs, all LMs between two consecutive respiratory events should be taken into consideration, especially in the absence of other periodic or patterned LMs during sleep. This is because it is not reasonable to score some of the LMs between two consecutive respiratory events as respiratory-related, while scoring the others as non-respiratory-related. This suggests a different pathogenesis between these LMs, but no factor other than the respiratory event seems to be related to the occurrence of LMs during the short and specific period of time between two consecutive respiratory events.

It is also possible that, in addition to RRLMs, there are other periodic or patterned LMs during sleep, especially during periods without respiratory events (e.g., Fig. 3). This suggests another potential pathogenesis behind LMs. To determine if and when the scoring of other periodic or patterned LMs, such as PLMs, should take priority over the scoring of RRLMs, or the other way around, the first question is whether or not there is a PLMS dominance. This means that, in some patients, compared with respiratory events, PLMS is a stronger or prioritized mechanism driving most or all LMs throughout the night. Thus, as suggested in previous studies that some PLMs may happen to synchronize with respiratory events [18], the occurrence of LMs is not related to respiratory events, even though some LMs may seem to be temporally related to respiratory events. This theorem is partially supported by previous findings that the number of PLMs increased or remained stable after successful OSA-related treatment [5, 21, 22], because OSA-related treatment cannot eliminate PLMs. However, in future studies, in addition to PLMS, which is a subdivision of LMs, attention should also be given to the change in the number of total LMs. Only when the number of total LMs remains stable after OSA-related treatment can it be confirmed that there is a PLMS dominance and OSA-related treatment does not affect LMs. In such studies, patients should be stratified based on AHI, which was reported to be associated with LMs [6, 23]. It is also because that, with the increase of AHI, respiratory events may play an increasing role in LMs and gradually become the dominant mechanism of LMs. In addition, it is also suggested to control for other LM-related factors, such as sleep stage, age, and gender [24, 25], which may further explain previous conflicting findings on the effect of OSA-related treatment on LMs, because even studies on patients with the same severity of OSA reported conflicting findings [21, 22]. If the presence of PLMS dominance is confirmed, further studies are needed to distinguish patients with PLMS dominance from others in order to facilitate treatment-related decision making. If no PLMS dominance is found and respiratory events always take priority over PLMS, then the authors would like to suggest to further delineate the time window to score RRLMs in a clinical evidence-based manner. This might be done by testing the effect of successful OSA-related treatment on the number of the recommended WASM criterion-based RRLMs and the number of PLMs and ILMs. If the number of PLMs and ILMs remains stable before and after successful OSA-related treatments, it means that most RRLMs are identified using the recommended WASM criterion. If not only the number of RRLMs but also the number of PLMs and ILMs decreased (not necessarily statistically significant) with successful OSA-related treatments, it suggests that many RRLMs are still mis-scored as PLMs or ILMs even based on the recommended WASM criterion. Then, further studies are needed to investigate whether LMs during respiratory events should also be scored as RRLMs. In this way, it can be demonstrated which LMs are truly respiratory-related and should be scored as RRLMs.

Previous studies found that LMs were frequent in the time window from -2.0 s to + 10.25 s around the end of respiratory events [13, 19]. In this study, we observed that many LMs during respiratory events were excluded from RRLMs based on this proposed time window. Although LMs during respiratory events are relatively rare, in future studies, attention should be given to the potential difference between LMs during respiratory events and those after respiratory events. Another point that needs to be discussed is arousal, which is imperative to RRLMs but was not taken into consideration in this study in order to keep this study focused. Previous studies found that RRLMs were more frequent when arousals were present, and the probability of RRLM doubled (from 26 to 64% [19] and from 23 to 53% [18], respectively) when an arousal was present at the end of a respiratory event. Another study on elderly men confirmed that RRLM% (viz., number of RRLMs/number of apneas and hypopneas) increased with the increase of arousal index [26]. However, it is still unclear whether arousals and RRLMs are directly associated with each other or whether their association is mediated by a shared provoking factor or mechanism. Further studies are needed to investigate the association among arousals, OSA events, and LMs, and to determine where the appropriate starting point is for addressing these complex and often co-existing issues.

This study has several limitations. First, to avoid excluding many participants due to insufficient LM and to avoid drawing conclusions based on chance events, 10 LMs of any type per hour of sleep was used as the best compromise for including participants in the present study. So, compared with previous studies [27, 28], this study included patients with more LMs and PLMs, and the findings may be limited to the current patient profile. Second, the effect of LM-related factors, such as age, gender, and arousal on the presence of LM was not investigated. Third, due to the reduced clinical capacity during the Covid-19 pandemic, only 32 participants were included in the study. The findings in this study should be confirmed in further studies with a larger sample size.

## Conclusion

LMs are more frequent after respiratory events than before and during respiratory events, and more RRLMs may be scored based on the recommended WASM criterion than based on the AASM criterion. However, to facilitate the diagnosis and treatment of LM-related conditions, more studies are needed to define the appropriate time window for scoring RRLMs.

## Data Availability

The datasets used and/or analyzed during the current study are available from the corresponding author on reasonable request.

## References

[1] American Academy of Sleep Medicine (2014). International Classification of Sleep Disorders.

[2] Ferri R, Fulda S, Allen R (2016). World Association of Sleep Medicine (WASM) 2016 standards for recording and scoring leg movements in polysomnograms developed by a joint task force from the International and the European Restless Legs Syndrome Study Groups (IRLSSG and EURLSSG). Sleep Med.

[3] Vetrugno R, D'Angelo R, Montagna P (2007). Periodic limb movements in sleep and periodic limb movement disorder. Neurol Sci.

[4] Telles S, Alves R, Chadi G (2011). Periodic limb movements during sleep and restless legs syndrome in patients with ASIA A spinal cord injury. J Neurol Sci.

[5] Budhiraja R, Javaheri S, Pavlova MK, Epstein LJ, Omobomi O, Quan SF (2020). Prevalence and correlates of periodic limb movements in OSA and the effect of CPAP therapy. Neurology.

[6] Kim HJ, Lee S (2020). Periodic limb movements during sleep may reduce excessive daytime sleepiness in men with obstructive sleep apnea. Sleep Breath.

[7] Szentkirályi A, Stefani A, Hackner H (2019). Prevalence and associated risk factors of periodic limb movement in sleep in two German population-based studies. Sleep.

[8] Covassin N, Neikrug A, Liu L (2012). Clinical correlates of periodic limb movements in sleep in Parkinson's disease. J Neurol Sci.

[9] Montplaisir J, Lorrain D, Godbout R (1991). Restless legs syndrome and periodic leg movements in sleep: the primary role of dopaminergic mechanism. Eur Neurol.

[10] Kapur VK, Auckley DH, Chowdhuri S (2017). Clinical Practice Guideline for Diagnostic Testing for Adult Obstructive Sleep Apnea: An American Academy of Sleep Medicine Clinical Practice Guideline. J Clin Sleep Med.

[11] American Sleep Disorders Association (1993). Recording and scoring leg movements. Atlas Task Force Sleep.

[12] Zucconi M, Ferri R, Allen R (2006). The official World Association of Sleep Medicine (WASM) standards for recording and scoring periodic leg movements in sleep (PLMS) and wakefulness (PLMW) developed in collaboration with a task force from the International Restless Legs Syndrome Study Group (IRLSSG). Sleep Med.

[13] Manconi M, Zavalko I, Fanfulla F, Winkelman J, Fulda S (2015). An evidence-based recommendation for a new definition of respiratory-related leg movements. Sleep.

[14] Berry RB, Quan SF, Abreu AR (2020). The AASM manual for the scoring of sleep and associated events: rules, terminology and technical specifications, version 2.6.

[15] Gurbani N, Dye TJ, Dougherty K, Jain S, Horn PS, Simakajornboon N (2019). Improvement of Parasomnias After Treatment of Restless Leg Syndrome/ Periodic Limb Movement Disorder in Children. J Clin Sleep Med.

[16] Littner MR, Kushida C, Anderson WM (2004). Practice parameters for the dopaminergic treatment of restless legs syndrome and periodic limb movement disorder. Sleep.

[17] DelRosso LM, Ferri R, Chen ML (2021). Clinical efficacy and safety of intravenous ferric carboxymaltose treatment of pediatric restless legs syndrome and periodic limb movement disorder. Sleep Med.

[18] Schipper MH, Alvarez-Estevez D, Jellema K, Verbraecken J, Fulda S, Rijsman RM (2020). Sleep-related leg movements in obstructive sleep apnea: definitions, determinants, and clinical consequences. Sleep Med.

[19] Fulda S, Heinzer R, Haba-Rubio J (2018). Characteristics and Determinants of Respiratory Event-Associated Leg Movements. Sleep.

[20] Homepage of plotly (n.d.). https://plotly.com/r/. Accessed 3 May 2023

[21] Baran AS, Richert AC, Douglass AB, May W, Ansarin K (2003). Change in periodic limb movement index during treatment of obstructive sleep apnea with continuous positive airway pressure. Sleep.

[22] Hedli LC, Christos P, Krieger AC (2012). Unmasking of periodic limb movements with the resolution of obstructive sleep apnea during continuous positive airway pressure application. J Clin Neurophysiol.

[23] Lee S, Lee Y, Cho C, Yang H, Im K (2021). Different scoring rules for respiratory event-related leg movements: effects on the prevalence of periodic limb movements during sleep and their association with depressive symptoms in patients with obstructive sleep apnea. Sleep Med.

[24] Haba-Rubio J, Marti-Soler H, Tobback N (2018). Clinical significance of periodic limb movements during sleep: the HypnoLaus study. Sleep Med.

[25] Szentkirályi A, Stefani A, Hackner H (2019). Prevalence and associated risk factors of periodic limb movement in sleep in two German population-based studies. Sleep.

[26] Aritake S, Blackwell T, Peters KW (2015). Prevalence and associations of respiratory-related leg movements: the MrOS sleep study. Sleep Med.

[27] O'Brien LM, Koo J, Fan L, Owusu JT, Chotinaiwattarakul W, Felt BT, Chervin RD (2009). Iron stores, periodic leg movements, and sleepiness in obstructive sleep apnea. J Clin Sleep Med.

[28] Lee SA, Kim SJ, Lee SY, Kim HJ (2022). Periodic limb movements during sleep are associated with poor health-related quality of life in patients with obstructive sleep apnea. Sleep Breath.

